# What incentives influence employers to engage in workplace health interventions? 

**DOI:** 10.1186/s12889-016-3534-7

**Published:** 2016-08-23

**Authors:** Camilla Martinsson, Malin Lohela-Karlsson, Lydia Kwak, Gunnar Bergström, Therese Hellman

**Affiliations:** Intervention and Implementation Research Unit, Institute of Environmental Medicine, Karolinska Institutet, Stockholm, Sweden

**Keywords:** Employers, Workplace, Incentives, Factors, Motivation, Occupational health and safety, Interventions, Health promotion, Disease prevention

## Abstract

**Background:**

To achieve a sustainable working life it is important to know more about what could encourage employers to increase the use of preventive and health promotive interventions. The objective of the study is to explore and describe the employer perspective regarding what incentives influence their use of preventive and health promotive workplace interventions.

**Method:**

Semi-structured focus group interviews were carried out with 20 representatives from 19 employers across Sweden. The economic sectors represented were municipalities, government agencies, defence, educational, research, and development institutions, health care, manufacturing, agriculture and commercial services. The interviews were transcribed verbatim and the data were analysed using latent content analysis.

**Results:**

Various incentives were identified in the analysis, namely: “law and provisions”, “consequences for the workplace”, “knowledge of worker health and workplace health interventions”, “characteristics of the intervention”, “communication and collaboration with the provider”. The incentives seemed to influence the decision-making in parallel with each other and were not only related to positive incentives for engaging in workplace health interventions, but also to disincentives.

**Conclusions:**

This study suggests that the decision to engage in workplace health interventions was influenced by several incentives. There are those incentives that lead to a desire to engage in a workplace health intervention, others pertain to aspects more related to the intervention use, such as the characteristics of the employer, the provider and the intervention. It is important to take all incentives into consideration when trying to understand the decision-making process for workplace health interventions and to bridge the gap between what is produced through research and what is used in practice.

## Background

Work-related injuries and illnesses are still a problem for many individuals, companies and societies around the world. It has been estimated that the economic costs of work-related injury and illness on a global basis vary between 1.8 and 6.0 % of gross domestic product (GDP) in country estimates, the average being 4 % according to the International Labour Organization ILO [1, 2]. In the United States it has been estimated that this costs approximately 250 billion US dollars a year on a national basis [3]. In Europe mental ill health and musculoskeletal disorders account for a significant share of work-related health problems [4–7]. In Sweden these disorders together account for more than 68 % of sick leave costs [6]. For the affected individual, both health problems and financial difficulties are evident [7, 8]. Beside the individual and societal consequences of work-related ill health, there are also consequences for the employer, such as increased costs related to absenteeism and presenteeism [9, 10].

One way to prevent work-related ill health and to promote health among the working population is through interventions targeting the workplace. Interventions which aim to prevent hazards arising in or from the workplace that could impair employee health and wellbeing are referred to as occupational safety and health interventions (OSH) [11]. Activities which aim to improve the health and wellbeing of people at work are referred to as workplace health promotion (WHP). OSH and WHP can be seen as complementary to each other and are both needed to achieve total worker health [12]. Current understanding of the effect of OSH and WHP interventions varies according to the targeted problem [13, 14]. However, research indicates that there are (cost-)effective ways to prevent ill health [15] and promote health [16] at the workplace. Although these interventions are available, there is still a gap between research findings and what is used in practice [17, 18], probably due to the difficulty of adapting research-based interventions to specific workplaces [18].

Legal requirements are important to encourage employer engagement in OSH and WHP. In Sweden these address the employer’s obligation to investigate, carry out and follow up activities in such a way that ill health and accidents at work are prevented and a satisfactory working environment is achieved. In a recently-published study from the Swedish Work Environment Authority it was shown that, despite legal requirements, many employers still do not fulfil their obligations [19]. This indicates that employers need additional incentives to increase their engagement in preventive workplace interventions.

A few international studies have investigated the employer perspective with regard to OSH and WHP interventions. In a British study it was found that legal requirements, economic incentives, moral and ethical aspects (in that order), were important incentives for managers in industry to spend money on employee health [20]. A Canadian study examined factors that explained managers’ intentions to increase discretionary spending on WHP programmes within the auto-parts industry [21]. In both studies the importance of so called “business cases” was identified as important for motivating managers to invest in employee health. Some of the managers maintained that their belief that WHP programmes reduce the indirect costs of health problems and their feeling of responsibility towards their employees was sufficient incentive to invest in such interventions. Similar results were found in another study conducted in the Canadian health care sector [22]. A literature review of employers’ motivation to carry out WHP found several factors to be important [23]. These were:

- Evidence of the economic benefits of WHP.
- Evidence of accidents and injuries as a consequence of poor employee health and wellbeing.
- Enhanced job satisfaction and commitment among workers, reduction of staff turnover and an improvement in the recruitment of new workers as a result of WHP.
- Evidence of indirect benefits, such as improved customer service and customer loyalty, as a result of WHP.

For workplace interventions to have an impact on employee health it is imperative that they are implemented in practice. To be able to support the implementation of workplace interventions, a good understanding is needed of the incentives which underpin employers’ use of OSH and WHP interventions. The aim of the present study is therefore to explore and describe the employer perspective regarding the incentives that influence their use of OSH and WHP interventions. To achieve this aim, focus-group interviews were held with representatives of a variety of employers and a content analysis was conducted of the transcribed interview material.

## Methods

### Definition of incentives

An incentive is a factor that stimulates a certain activity. It can be applied to groups as well as individuals. It can be intrinsic, extrinsic and negative or positive in nature [24–26]. Incentives can differ from one group or person to another, from one situation to another, all depending on the value the group or person places on those incentives at the time [25]. Intrinsic incentives refer to an action that is performed for its own value, for example joy or excitement. Extrinsic incentives, on the other hand, refer to an action taken in order to obtain or avoid an outcome, for example to obtain improved productivity or to avoid reduced productivity at a workplace. People are drawn towards behaviours that offer positive incentives and repelled by behaviours associated with disincentives [24]. In this study the term incentive is used to refer to a factor that stimulates a certain activity, i.e. the incentives which influence an employer’s use of OHS and/or WHP interventions.

### Design

In this study a qualitative design with semi-structured focus group interviews has been applied. The focus group interview method is based on the conception that the interaction between responders, as opposed to having only one respondent, raises their awareness and ability to explore and clarify individual and shared perspectives [27]. This research design was chosen because of the pre-existing lack of knowledge regarding the objective of the study [18, 20–22, 28] and our interest in gathering a broad range of information about it. The study has been reviewed by the Swedish ethical review board, who determined that the research does not involve the processing of personal data referred to in the Ethical Review Act and is therefore not covered by the regulation. For this reason the ethical review board deemed that the study did not require ethical approval (reference no 2014/58-31/5).

### Participants

The study population consisted of managers from a variety of economic sectors in Sweden with responsibility for making decisions about OHS interventions or other employees directly involved in this work with a mandate to answer questions about these decisions. The inclusion criteria for the participants were: working in a medium or large company and having been at their current company for least 6 months. Small employers were deliberately excluded because their conditions and prerequisites look essentially different from those of medium and large businesses. For example, existing OSH or WHP programs are often too costly or time consuming for small employers [29]. These employers also often lack a formal department or a staff that is responsible for occupational safety and health at the workplace [29]. Purposive sampling was used to include participants in the focus groups because this sampling method aims to target individuals who have experience of and can offer specific information to the researchers regarding the objective of their study [30]. Using this method the participants were selected one after the other to fill the focus groups and to create a variation in type of employer and economic sector. The participants were located through informational internet websites, work related contacts and snowballing.

About 170 people were located and informed about the study by e-mail. In the e-mail they were offered the opportunity to have the focus group interview either in their own town or nearby, in order to facilitate participation. They were also told how long the interview would take. Participants were not offered any economic incentive to participate, nor any goods or gifts. If the contacted person did not respond to the e-mail, or if they asked to be called back, they received a follow-up phone call. Approximately 40 persons were interested in participating but 20 were unable to participate for reasons such as not being able to participate in such a long interview because of workload, a sudden impediment the day of the interview and/or being unavailable on the same dates as any of the other participants in the focus groups.

Twenty participants gave their written informed consent and attended the focus group interviews. The participants were informed that they could withdraw from the study at any point in time without stating any reason. Five groups were formed ranging in size from two to five participants. One participant had worked at her current workplace for less than 6 months but her total experience of working with the issues in question was 10 years. The participants represented employers who, together, employed people throughout the country. However, the focus group participants were mainly located at their organisations’ head offices, located near two large cities in central Sweden. The economic sectors represented in the focus groups were municipalities, government agencies, defence, educational, research, and development institutions, health care, manufacturing, agriculture and commercial services. The participants had the following work titles: chief executive officer (CEO) (*n* =1), staff executive (*n* =2), occupational health executive (*n* =1), human resources (HR) executive (*n* =5), HR business partner (*n* =1), HR specialist (*n* =4), occupational health and safety specialist (*n* =2), health strategist (*n* =3), and staff administrator (*n* =1). Table 1 gives an overview of the characteristics of the study participants.

**Table 1:** Characteristics of the participants in the study

Variable	All participants (*n* = 20)	Focus group 1 (*n* = 4)	Focus group 2 (*n* = 6)	Focus group 3 (*n* = 5)	Focus group 4 (*n* = 3)	Focus group 5 (*n* = 2)
Gender *(female/male)*	17/3	3/1	6/0	4/1	2/1	2/0
Sector *(private/public)*	12/8	3/1	3/3	5/0	1/2	0/2
Years of total working experience with OHS						
*Mean (SD)*	15.50 (9.2)	14 (9.8)	13.8 (9.9)	17.2 (9.3)	10.7 (3.8)	27 (9.9)
*Range*	2–34	2–26	4–27	9–30	8–15	20–34
Years of working experience at current employer and position						
*Mean (SD)*	6.40 (4.5)	9.6 (1.5)	6.4 (2.4)	4.75 (2.6)	6.7 (2.9)	10.5 (13.4)
*Range*	0.29–20	0.5–11	1.5–9	0.29–7	5–10	1–20
Number of employees at the represented workplaces						
*Mean (SD)*	4780.50 (4747)	3912.5 (3275.8)	4650 (4156.3)	6190 (6655.9)	1870 (2296.7)	5000 (4808.3)
*Range*	260–17,000	650–8000	500–11,000	350–17,000	260–4500	1600–8400

### Data collection

The focus group interviews were held in two large cities in central Sweden between March and September 2014. Alternative locations and dates were offered to facilitate participation. All interviews were semi-structured [30], meaning that they were prepared with an interview guide with a pre-determined set of open questions, setting a frame that allowed new information to be brought up with the aim of exploring the objective of the study [30]. The interview guide included questions regarding incentives for OHS and WHP interventions, decision making, and experiences relating to the subject. Examples of questions:

*“What incentives have you had at your workplace when choosing to engage in OHS or WHP interventions?”*

*“What incentives affect the decision to use one OHS or WHP intervention over another?”*

Before the start of the interviews all participants were once again told about how the research group handle the data and keep it secure and confidential. Participants were also told that they could not be prevented from talking about what had been said in the interviews. However, they discussed the confidentiality of the information in the interview before the session started and agreed to keep everything in the interview sessions to themselves. They signed an informed consent form and a short descriptive demographics and background form. The participants and the researchers also had breakfast or lunch together before the interviews to make the group feel more comfortable and relaxed with each other [30]. Each interview lasted between 83 and 124 min. According to the principles for conducting focus group interviews, they were conducted by two researchers, one being responsible for moderating and the other for observing [30]. The interviews were digitally recorded and transcribed verbatim.

### Data analysis

A latent content analysis was used to perform an explorative inductive analysis of the data [31]. The analysis was conducted in the following way: 1) All transcribed focus group interviews (221 pages) were read through several times to get to know the content and obtain a sense of the whole; 2) The content or data in each focus group interview that related to the objective were highlighted and condensed into meaning units, i.e. the highlighted text was summarised into shorter notes; 3) The condensed meaning units were then abstracted, i.e. interpreted regarding explicit meaning and/or possible underlying meanings and given codes, i.e. a title relating to the interpretation; 4) The condensed meaning units and codes for each transcribed focus group interview were then listed in separate MS Word documents to see whether the condensed meaning units and codes within each separate document were linked to each other and focused on the same thing; 5) The related codes within each separate document were then organized and merged into categories and sub-categories, see Table 2 for an example of categories and sub categories; 6) All the interviews were then compared with each other to see if the categories and subcategories were linked to each other and focused on the same thing. The ones that did were merged together, resulting in five categories and nine sub-categories. During the whole procedure described above the researchers went back and forth reading the transcribed interviews to make sure that the results did not lose their meaning in relation to the original context. Table 2 gives an overview of the analysis.

**Table 2:** Over view of the steps in the content analysis

Meaning unit	Condensed meaning unit - Description close to the text	Condensed meaning unit - Interpretation of the underlying meaning - Code	Sub-category	Category
Our goal was to reduce the number of sickness absence days, so that the employees return earlier from their sick leave. This has a lot to do with money of course.	Interventions to reduce sickness absence days have to do with money.	Interventions to avoid costs.	Preventing negative consequences.	Consequences for the workplace.
It is the fact that sickness absence, dysfunctional employee groups and staff turnover cost money.	The problems that cost money for the workplace lead to interventions.			
I would like, in a stressful situation, to have suggestions from them (the suppliers). It is absolutely impossible to think of those high-quality solutions, you need suggestions, so you can pick and choose. But often they are unable to deliver proposals, it stops well before the proposals. It stops too early.	Many suppliers do not manage to deliver proposals for additional interventions, proposals would give incentives.	Other aspects then those relating to the intervention itself gives incentives.	Feedback.	Communication and collaboration with the supplier.
It is possible that this is a desire that’s unclear from us… That we must address this further in order for the suppliers to come back with suggestions for solutions (after *e.g.* health surveys). It is an excellent opportunity for them to sell.	To increase incentives for OHS interventions the communication needs to be better between the byer and the supplier.			

The analysis was conducted by two researchers to strengthen the reliability of the analysis and minimize the risk of the analysis being characterized by one person and his or hers possibly own understanding of the phenomenon [31]. One person performed the above-mentioned steps on all the interviews. The other person performed the same steps on the first interview, in addition to reading and asking new questions regarding the rest of the material, and presented alternative ways to interpret and understand the data. In a later phase of the analysis process, two more persons were involved in reading the material and asking probing questions about it, to ensure that the analyses would stand scrutiny. The research team was inter-professional and represented a variety of different experiences and perspectives. The reason for using an inter-professional team was to further strengthen the reliability of the analysis and minimize the risk of the analysis being characterized by a certain professional background [31].

## Results

The findings describe incentives that influence employers’ use of OHS and WHP interventions. A variety of incentives was identified in the analysis. The incentives seemed to influence the use of interventions parallel with each other and were not only related to positive incentives to engage in OHS interventions, but also to disincentives affecting the employer’s decision to initiate workplace health interventions. For some of the participants, the incentives were clear and well thought through, other participants had only briefly reflected upon them. The incentives identified in the analysis have been divided into five categories and nine sub-categories.

### Laws and regulations

Laws and regulations were described as an incentive influencing the use of OHS interventions. OHS interventions that are stated in law and/or mentioned in regulations were always implemented, partly because it was considered mandatory to follow the law, but also to avoid conflicts with trade unions as described by one participant:

*“To comply with the Work Environment Act, is perhaps the most important incentive, and not to upset the union”* (Focus group 5)

The participants regarded laws and regulations as important incentives because employers generally intended to follow them, which also made it easier to justify them and obtain stakeholder agreement and financial support for these interventions. Some participants stated that their managements were satisfied when these demands had been fulfilled. However, participants often felt that the interventions supported by law were seldom sufficient and that further interventions were needed. One of the participants said the following:

*“Everything that has legal support when it comes to rehabilitation and work environment rolls on, well, we are home here… But it’s the other parts that are limping”* (Focus group 2)

### Consequences for the employer

Participants stated that the prevention of negative consequences and the promotion of positive consequences for the employer served as incentives which influenced decisions. The prevention of negative consequences was, for example, avoiding unnecessary costs, while the promotion of positive consequences was mentioned in terms of improved production, sustainability and other non-specified benefits for the employer. This is explained further in the two sub-categories “*Preventing negative consequences*” and “*Promoting positive consequences*”.

#### Preventing negative consequences

Participants said that short-term and long-term sickness absence resulted in high costs for the employer and therefore constituted an incentive for decisions to engage in OHS interventions. One participant described the following attitude:

*“Our goal has been to reduce the number of sickness absence days, so that the employees return earlier from their sick leave… This has a lot to do with money of course”* (Focus group 2)

Participants also considered it important to avoid future costs by paying attention to employees at risk of repeated short-term or long-term sick leave, which workplace health interventions were seen as a way of trying to prevent. One participant said: *“The incentives at our workplace are the economic aspect, that sickness absence costs money, dysfunctional employee groups cost money, staff turnover costs money”* (Focus group 2)

#### Promoting positive consequences

Participants described that workplace health interventions which had positive consequences for the employer constituted incentives to engage in them. The positive consequences referred to by participants were varied and more or less specific. They included improved production, improved revenue, sustainability of the workplace and non-specified benefits for the employer. Participants referred to three types of improved production: improved efficiency, improved quality and improved productivity. Sustainability of the workplace was described as having sustainable employees with the energy to perform optimally both at work and in private life, being able to cope with occasional stress and periods of higher work load. Non-specified benefits for the employer were expressed as those which, beside improved employee health, arise as a result of involvement in OSH interventions. The type of positive consequences achieved were not specified or always known.

Improved production and sustainability of the workplace were often mentioned together, but not always. For example, having a sustainable workplace could be seen as a single incentive, but it could also be seen as a factor contributing to other incentives such as improved production. The following quote describes some of this:

*“Why we want to invest in this (OSH interventions) is because we know that we get more productive employees of course. If we for example talk about exercise and physical activities we know that it buffers against negative stress. Sustainable employees are how we think now a days…”* (Focus group 3)

Participants also mentioned improved production and/or increased revenue as incentives, without mention them in relation to sustainability:

*“You must have some form of wellness activity … you have to be healthy on the job, that’s what they want … They are not interested in anything other than that people come to work and produce”* (Focus group 1)

In other cases, the participants were more or less clear and specific about the positive consequences. For example, they wanted their employees to enjoy being at work and to feel that the workplace contributed to a feeling of wellbeing. Some participants clearly stated that they believed that increased wellbeing contributes to several positive side effects. This was described as follows by one participant:

*“The thing is that we are always trying to work in this direction (in relation to OHS-interventions)… It should be fun to go to work, people should feel that they want to go to work. There are people who feel so bad at work that they might be sick a little more, you might say. The threshold to go to work is a little higher. If I feel a little under the weather I might stay home”* (Focus group 4)

OSH interventions were also seen as a way of strengthening the brand as an attractive employer and attracting the best employees in order to create satisfied customers. Others said that they wanted to improve employee wellbeing, believing that it would lead to some sort of benefit for the employer, even if the exact benefit was unknown.

*“There has been a proactive dialogue on these issues for many years in the management of the company, I can’t see any other explanation for us having built up such a strong health center. It is not based on deviations, it is based on working with health. There is an idea about doing something that is good, without necessarily measuring it in monetary terms”* (Focus group 4)

### Knowledge of worker health and workplace health interventions

The participants mentioned various types of knowledge as important incentives. This knowledge was often based on their own experiences of, for example, physical activity in relation to health or the availability of certain OSH interventions. However, this knowledge was not necessarily evidence-based. The different aspects of knowledge described by the participants are elaborated on in the following two sub-categories: “*(Lack of) Knowledge of worker health and workplace health interventions*” and “*(Lack of) Knowledge of worker health and workplace health interventions on the part of the provider*”.

#### (Lack of) Knowledge of worker health and workplace health interventions

Most of the participants who said that economic and/or other benefits for the employer served as incentives influencing decisions about OHS interventions had prior knowledge about the consequences of ill health and the benefits of good health for the employer. They also knew that some of the consequences could be promoted and prevented with the help of workplace health interventions. This knowledge could be derived in several ways, for example through colleagues or personal experience:

*“If the management takes exercise and has a healthy life style, they will spend more money on health related activities at work. I think that’s one of the reasons why we have a health center at our company. That there is a deeper belief in the management that this is something good… They have seen the benefits of exercise and a healthy life style throughout life… You work … a little better if the body is fit, you can aim a little higher when necessary and also unwind more easily, they’re linked to each other”* (Focus group 4)

Other participants said that neither they nor others at their workplaces had the above-mentioned knowledge or experience. These participants saw this knowledge as difficult to access and did not know where to obtain it. They said that due to the lack of the above-mentioned knowledge they or others at their workplace often took the decision to engage in workplace health interventions without any clear thoughts about it. These decisions could be influenced by, for example, a phone call from a salesman, an acquaintance, colleague or another person who had said that an intervention was good without substantiating why; other employers who had used a certain type of intervention; or trends in workplace health interventions. One participant responded as follows to the question about what kind of incentives determine which workplace health intervention are engaged in:

*“Really, it’s hard to specify what it is that influences and affects what we are deciding on. We are probably a little bit like - Oops, there’s an intervention! Do we have money? Yes, we do. We check some references. Let’s go for it!”* (Focus group 2)

#### (Lack of) Knowledge of worker health and workplace health interventions on the part of the provider

Participants stated that specialist knowledge or skills on the part of the providers gave them incentives to engage in workplace health interventions. The participants said that they were not interested in engaging in workplace health interventions given by providers who delivered both organizational and individual level interventions. They argued that it is hard to be good at everything within these interventions and that they had greater confidence in those who were specialized in a certain field. As an example, participants stated that they had less trust in providers who had historically provided medical examinations and healthcare when it came to leadership training and organizational development. Such doubts could have a variety of consequences depending on what employer the participants represented. For example, participants from the public sector were often tied to contracts and/or tight budgets without the freedom to pick and choose between different providers for every new workplace health intervention. This situation could sometimes cause participants to refrain from interventions that they did not believe their providers were expert enough to perform satisfactorily. Participants who worked in the private sector could generally choose which provider they preferred and discard those they believed lacked specialist skills. One participant from the private sector expressed in the following way:

*“When you buy something you want it from a provider who’s specialized and absolutely the best in the market at the time. You don’t want a provider of everything. When X occupational health service is trying to sell leadership training to me, I think, oh well. Why? Forget it! Then I’ll go out and look at the top three providers of leadership training and choose one of them.”* (Focus group 3)

An important incentive was that providers not only have specialist knowledge but are also up to date on new research. However, it was commonly felt that providers seldom updated their services in accordance with new research evidence. The participants pointed out that research and general knowledge become outdated over time. Not adapting to the latest findings erodes trust in a provider’s ability to perform effective workplace health interventions, resulting in less interest in using their interventions. One participant said the following about what gives an incentive to engage in, or not to engage in workplace health interventions:

*“I think of occupational health services … What I feel is that they’re still in the 70s. If they had kept up and were more in touch with today’s organizations and employers … The collaboration between us and them could have been different and we could have met in another way”* (Focus group 5)

### Characteristics of the intervention

Participants described that how they perceived the characteristics of an intervention was important in influencing whether to engage in workplace health interventions. The characteristics of an intervention were identified as the following three sub-categories: “*Evidence based research or successful examples*”, “*Measurable effects*” and “*Easy to perform and easy to understand*”.

#### Evidence based research or successful examples

Proof that the intervention was effective, as demonstrated either by research results or examples from other employers, was an important incentive. One participant said that her workplace only focused on the health areas for which evidence based research into workplace health interventions was available. Another participant’s workplace had chosen to stop using an intervention since no evidence of its effect was available. A third participant said the following:

*“What works as an incentive is if you know that other workplaces that have used a workplace health intervention and have achieved results. This is something that works”* (Focus group 1)

#### Measurable effects

Another incentive reported by the participants was the ability to measure the outcome of a workplace health intervention after implementing it. It was seen as important that the effect evaluation was designed by the developer or provider of the intervention before implementation. This was important in order to be able to justify the use of interventions. One participant described this as follows:

*“If we want to gain acceptance (from the rest of the management) to work with health, we must be able to measure what effects the interventions provides …”* (Focus group 4)

They also said that measurable outcomes were important because the management often asked about effects after as little as one month; the quicker the results show the better. The demand for measureable outcomes sometimes created problems with regard to interventions designed to improve, for example, the psychosocial work environment, which can be difficult to measure. This was suggested as an explanation for fewer interventions of this kind being carried out.

#### Easy to perform and easy to understand

The participants said that interventions that are easy to adopt gave incentives to engage in workplace health interventions. A decision to engage in an intervention was seldom made if it was unmanageable, required comprehensive routine changes, contained a large amount of information, was difficult to understand and time-consuming. According to participants, the reason for this was that managements generally prioritize activities that directly target the main goals of the workplace over other activities, such as workplace health interventions. If an activity that does not directly contribute to the goals of the workplace takes time and energy from this work, it will be terminated or not engaged in. One participant gave the following response to the question about which incentives influence the decision to engage in a workplace health intervention:

*“What puts a spanner in the works is the practical situation. What do we have time for? Can they (the employees) go away? When there are things to be done that are not directly woven into the job, then time is a big factor”* (Focus group 5)

### Communication and collaboration with the provider

The participants brought up communication and collaboration with the provider as important incentives. Communication and collaboration were described as the provider’s interest in adapting to the workplace goals, needs and culture. It was also described as the provider’s way of giving feedback to the workplaces. The following two sub-categories describe this further: “*Responsiveness and adaption to the workplace goals, needs and culture*” and “*Feedback*”.

#### Responsiveness and adaption to the workplace goals, needs and culture

It was seen as important that providers should first perform a customer analysis and then provide a tailored intervention. The participants expressed frustration over the fact that providers often offer universal solutions and predefined concepts. If interventions were tailored to the specific workplace’s needs and prerequisites, there would be a much greater incentive to engage in the interventions because management could feel more assured about their effectiveness. The following quotes are from three interview participants in a discussion about what makes them choose one provider over another:

- *Participant 1: “That they can do what we want and not just sell a concept.”*
- *Participant 2: “They must understand the company’s strategy. Understand the company’s values. They must also make an effort to get to know the company, rather than just say ---”*
- *Participant 3: “This is what we offer!”*
- *Participant 2: “Exactly!”* (Focus group 3)

#### Feedback

The participants identified feedback from the provider as an important incentive. They talked about feedback in relation to how the providers presented their results during and after interventions and how they worked with confidentiality. It was important for them to know in advance how the providers intended to convey feedback to them. They wanted providers who gave concrete suggestions for solutions to identified problems during and after interventions and who do not use more confidentiality then necessary. Participants reported that they had rejected or chosen to end their collaboration with providers who had not been able to meet their needs in this regard.

Receiving suggestions for how identified problems could be solved were important incentives for further workplace health interventions. Frustration was expressed about only receiving information about existing problems, without suggestions for how to prevent these problems:

*“In a stressful situation I would like, to have suggestions from them (the providers). It’s absolutely impossible to hit upon those high-quality solutions yourself, you need suggestions, so you can pick and choose. But they are often unable to deliver suggestions, it stops well before getting to a suggestion. It stops too early”* (Focus group 1)

However, the participants were also aware that they needed to become better at informing the providers that they wanted this kind of feedback. They were aware that the lack of communication was a mutual problem:

*“It’s very possible that we are unclear… That we must address this further in order for the suppliers to come back with suggestions for solutions (after e.g. health surveys). It is an excellent opportunity for them to sell”* (Focus group 1)

The participants took up the issue of confidentiality, when the providers withheld information about individual employees and only presented information and results at group level. They experienced that the providers often held back more information than was necessary and thus made it difficult for the employers to decide about how to do follow-ups and further interventions. They saw this as a problem because it prevented the employers from taking their responsibility for their employees. Two participants said the following in a discussion about this:

- *Participant 1: “If we have a survey amongst the employees and the statistics shows that we have 15 people who feel harassed or bullied. We do not know who they are and the providers say something like “We won’t disclose that!” and there are 250 of us in the workplace.”*
- *Participant 2: “I cannot see any other industry where you end up in such a dilemma. We have information that there is something crazy going on, we have a provider who’s supposed to help us with it, but won’t disclose the information and there is nothing we can do about it.”* (Focus group 1)

## Discussion

This qualitative study explored and described the employer perspective regarding the incentives which influence their use of preventive and health promotive workplace interventions. Several incentives were identified and some are to a certain degree consistent with those from other studies, namely the two categories “laws and regulations” [20, 22] and “consequences for the employer” [20–23] and the two sub-categories “(lack of) knowledge of worker health and workplace health interventions at the workplace” [22] and “evidence based research and successful examples” [20–23]. Additional incentives that emerged in our study (and have not already been mentioned here, or only briefly in the review of employers’ motivation for carrying out WHP [23]), were the category “communication and collaboration with the provider” and the sub-category “easy to perform and easy to understand”.

Previous research has concluded that even though several research-based interventions are available, there is still a gap between what is produced through research and what is used in practice [17, 18]. The findings of the present and previous studies [23] describe a large variety of incentives regarding workplace health interventions. The present study indicates that employers take a number of incentives into account when deciding about engaging in workplace health interventions. The decision to engage in interventions is proven to be complex and multifaceted, with incentives linked to both the employer’s and the provider’s previous knowledge and ability to communicate with each other as well as the characteristics of the interventions. These findings suggest that in order to bridge the gap there is a need for a broad approach that includes adjustments from the employers themselves, from providers and from OSH researchers.

In both this and previous studies, laws and regulations have been identified as strong incentives affecting employers’ engagement in workplace health interventions [20, 22]. Our findings revealed that these were strong incentives, partly because employers feel a duty to abide by the law. Interestingly, the participants experienced these incentives as both a facilitator for and as a barrier to engaging in workplace health interventions. Legislation made it easier to justify and obtain stakeholders’ agreement and financial support for workplace health interventions. However, participants also described the law as a barrier since they felt that only doing as much as required by the law was not nearly enough to achieve a good work environment, while the management was often satisfied with this. Having laws regulating the work environment is good but they need to be complemented by other incentives to increase the use of workplace health interventions.

The findings indicate that employers are aware of the need to focus on workplace health interventions that are research-based and proven to be effective in relation to desired outcomes at the workplace, which is in line with previous research [20–22]. On the other hand, our findings also reveal that interventions are sometimes chosen based on others’ experience or a chance telephone call from a salesman. Furthermore, the participants had difficulty accessing sufficient information about workplace health interventions and their expected outcomes, which might in part explain the wide variety of factors which influenced their decisions. They also stated that a thorough customer analysis conducted by the workplace health providers was often not available. This lack of information could be interpreted not only as a consequence of insufficient communication and information from workplace health providers and researchers, but also as a consequence of the employer’s limited ability to assimilate given information. The latter problem has been identified in previous research [22], but then primarily in relation to economic methods of evaluation of workplace health interventions. The findings from the present study do not elaborate on this and further research is needed regarding communication and the ability to assimilate information on the part of the different stakeholders. Some clinical implications can nonetheless be drawn based on our findings and their possible interpretations. In order to increase the use of evidence-based workplace health interventions, it might be important for the providers to first perform thorough, customized analyses. Furthermore, it might be beneficial to help employers’ access research into workplace health interventions by developing proper guidelines for such interventions based on current research evidence. Helping employers with this could help them to understand the interventions better, something that has also been identified in the present study as an incentive. The identified incentives are related to health promotive and preventive workplace interventions in general, rather than to specific interventions. It might however be the case that incentives differ between different types of intervention. This implies that some incentives (i.e. employers’ preferences) are only important in decisions about certain types of intervention. Further studies are needed to deepen our understanding of the role of different types of incentive for different types of intervention.

The findings of the present study are in line with existing theoretical frameworks within implementation research that are used to identify barriers to and facilitators for implementing evidence-based interventions into practice. Future studies should focus on applying an implementation framework such as the Consolidated Framework for Implementation Research as a guide for systematically structuring interviews aimed at assessing potential barriers to and facilitators for implementing workplace health interventions. This would facilitate a more in-depth understanding of the theoretical constructs that can influence an employer’s decision to implement interventions of this kind.

### Limitations

The goal of this study was to hold focus group interviews with 4–6 participants since this is recommended in the literature [27]. This number of participants was unfortunately not achieved in two of the focus groups, which can be seen as a limitation. The groups were constructed to include four or more participants, but the actual numbers varied because of late cancellations. Because of the difficulty in coordinating the participants and bringing them all together for the interviews, it was decided to carry out the focus group interviews even though only two or three participants attended. This may have affected the desired interaction between the participants, although not necessarily. Having smaller focus groups can also be a strength when participants are expected to have much to say about the topic [30]. The participants in this study were purposely sampled with specific inclusion criteria set to support in-depth answers and dialogues about the objectives of the study.

One participant did not fulfil the inclusion criteria because she had worked for less than 6 months at her current workplace. This came to our knowledge just before the interview session started and can be seen as a limitation. However, she had worked with the issues in question for 10 years in the same economic sector. The aim of this inclusion criterion was to make sure that the participants had enough experience to have something relevant to say in relation to the objective of the study; the authors reasoned that this participant, given her long experience of working with these issues, made her relevant for the study with much to contribute to her focus group.

The fact that we invited a large number of participants and only a few agreed to participate may have had a positive influence on the results because the participants who did attend may have been unusually interested and seen more reasons for engaging in workplace health interventions than those who chose not to participate. However, several potential participants declined to participate because of heavy work load and limited time. The study also included participants who had few workplace health interventions in place and/or little knowledge about these interventions, thereby lessening the concern that only participants with prudent and extensive workplace health interventions participated.

It would have been beneficial to perform a member check in order to further enhance the trustworthiness of the study. However, this was not possible for practical reasons, such as participants finding it difficult to spend even more time on the study and the considerable length of time between data collection and analysis.

## Conclusions

This qualitative study into the incentives which influence employers’ use of workplace health interventions suggests that the decision is not influenced by one incentive alone but by several in combination. Our study revealed that employers were often satisfied when basic legal requirements had been met. However, knowledge of workers’ health and workplace health interventions was also one important incentive, although often based on personal experience and not necessarily on research evidence. Furthermore, communication between the employer and the provider was not always seen to be satisfactory, a factor which influenced the decision about whether to engage in an intervention. To facilitate the implementation of workplace health interventions there is a need to develop guidelines which specify the important incentives to take into consideration when evidence-based workplace health interventions are being developed. It would also be beneficial to provide a checklist of important aspects that need to be communicated in the collaboration between the stakeholders.

## Abbreviations

EU-OSHA: European agency for safety and health at work
GDP: Gross domestic product
ILO: International labour organization
OSH: Occupational safety and health
SWEA: Swedish work environment authority
WHP: Workplace health promotion
